# Frequency of allele variations in the *CFTR* gene in a Mexican population

**DOI:** 10.1186/s12920-021-01111-w

**Published:** 2021-11-05

**Authors:** Consuelo Cantú-Reyna, Roberto Galindo-Ramírez, Mercedes Vázquez-Cantú, Lorenza Haddad-Talancón, Willebaldo García-Muñoz

**Affiliations:** 1 Laboratorio de genética humana, Código 46, S.A de C.V, Camelias 3-Int 10, Los Tabachines, C.P. 62498 Cuernavaca Morelos, Mexico; 2 Escuela de Medicina y Ciencias de la Salud, Tecnológico de Monterrey, Monterrey, Nuevo León Mexico

**Keywords:** Allele frequency, Cystic fibrosis, *CFTR* gene, CF variants, CFTR-RD variants, Pathogenic and likely pathogenic variants

## Abstract

**Background:**

Cystic fibrosis (CF) is an autosomal recessive disorder caused by pathogenic variants in the cystic fibrosis transmembrane conductance regulator (*CFTR*) gene. The CF variants incidence is highly variable and even undetermined in some countries like Mexico.

**Methods:**

In this study, the allele frequencies of 361 variants in the *CFTR* gene were investigated in 1455 Mexicans without a CF or *CFTR*-related disorders (CFTR-RD) diagnosis. We also performed a statistical comparative analysis against allele frequencies of different populations to measure genetic differences in the prevalence of *CFTR* variants.

**Results:**

In the vast majority of cases, the allele frequencies of this cohort were comparable to those found in other populations. However, some variants displayed significant differences in their allele frequencies when compared with European and African populations.

**Conclusions:**

This study provides information about *CFTR* variants to predict the prevalence of CF in Mexico and uncover other unknown but frequent pathogenic variants in the country. Additionally, other CFTR-RD variants have also been studied using population data of the same *CFTR* variants. Studies like this could help develop a regional molecular diagnostic screen to optimize the medical care of CF patients.

**Supplementary Information:**

The online version contains supplementary material available at 10.1186/s12920-021-01111-w.

## Background

Cystic fibrosis (CF) is considered a rare disease, with an estimated prevalence of 70 to 100 thousand affected individuals worldwide. It is one of the most common autosomal recessive disorders [1]. This condition is caused by genetic variants in the *CFTR* gene, which encodes a protein that functions as an anionic channel involved in water and chloride ion homeostasis [2]. Pathogenic variants in this gene cause an accumulation of thick and sticky mucus in the lungs, pancreas and intestines that generally lead to severe complications. Initial symptoms frequently start in early infancy and include gastrointestinal disorders like meconium ileus, liver disease, intestinal obstruction and pancreatic insufficiency that result in malnutrition, developmental disorders and diabetes mellitus [3, 4]. Lung disease is the main cause of morbidity and mortality in patients with CF. Isolated manifestations, called *CFTR*-related disorders (CFTR-RD), such as congenital absence of vas deferens, idiopathic chronic pancreatitis, bronchiectasis, among others could be present as well. CF affects 1 in 3500 newborns worldwide with a higher disease prevalence in Caucasian and Ashkenazi Jews populations [1, 5]. The Mexican Association of Cystic Fibrosis and other research groups estimate a disease prevalence in Mexico between 1 in 5000–8500 newborns [6–8].

According to dbSNP, ClinVar and Cystic Fibrosis Mutation Databases [9, 10], there are more than 2000 described variants in this gene. However, only a fraction of them are pathogenic [10]. The greatest number of CF cases worldwide are caused by the most prevalent pathogenic *CFTR* variants [1, 11] and it is reported that NC_000007.13:g.117199646_117199648del is the most frequent CF pathogenic variant in Caucasian populations. Although the reported spectrum and frequency of *CFTR* variants varies by country and ethnicity [1, 5, 12]. For instance, the NC_000007.13:g.117246808G>T variant, while uncommon in Caucasians, is the second most frequent CF allele among African individuals, occurring at a frequency of 10 to 12% [5]. In fact, according to the World Health Organization Humans Genetic Programme, 25% of the *CFTR* alleles of CF patients are rare and unique to each population [1, 5, 12, 13]. Which suggests the need for regional studies of molecular diagnostics screens to optimize medical care for CF patients [5, 6, 14].

In general, the study of innate errors of metabolism represents a challenge in public health, and currently neonatal metabolic screening programs are the only strategy allowing the early detection and intervention of this group of conditions. In Mexico, the detection panel through the neonatal metabolic screen varies from 1–6 diseases in the public health sector and 1–76 diseases in the private sector. CF is considered a rare disease of primary concern due to its high prevalence, and complementary molecular studies such as genotyping and next-generation sequencing have been proposed as confirmatory tests when a newborn is detected with a probable diagnosis of CF. To date, knowledge of the spectrum of *CFTR* variants in the Mexican population has remained limited. To determine which *CFTR* alleles are prevalent in the open Mexican population, we analyzed microarray genotype data of 1754 Mexicans without a CF or CFTR-RD diagnosis. The results of our study could be used to design proper screening panels of CF variants in our country [14, 15]. We also performed a comparative statistical analysis of *CFTR* allele frequencies against other world populations. This study offers an epidemiological outlook of CF in the Mexican population, with an accurate estimation of the frequency of several genetic variants in the *CFTR* gene.

## Methods

### Database of genetic variants of a Mexican population

We performed a retrospective analysis of genetic variants collected between May 2017 and May 2019 from the human genotyping program of Código 46, a Mexican company that provides human genotyping services and conducts research in the field of human genetics for the development of personalized medicine in Latin America. During this project, human cells were collected from buccal smear samples using a specialized swab (4N6FLOQSwabs, Thermo Fisher Scientific) and the genomic DNA was extracted using an in-house modified salting-out method. Briefly, the swab tip was suspended in 500 µL of lysis solution containing 10 mM Tris, 500 mM EDTA, 10% SDS, 40 mg/mL proteinase K (Thermo Fisher Scientific) and incubated at 55 °C for 1 h. DNA was precipitated by adding sodium acetate 3 M and isopropanol. DNA concentration and integrity were assessed using spectrophotometry at 260 nm and electrophoresis with a general-purpose agarose E-Gel (Invitrogen). The samples with 30–50 ng of DNA were genotyped with the Illumina Infinium HTS Automated protocol and the Beadchip Global Screening Array (GSA-24 v1.0) microarray. The genotype calling was performed using the Illumina GenomeStudio Genotyping software (Illumina, USA) and only the samples with a call rate greater than 0.95 were considered for this study. The whole protocol was performed in Código 46’s laboratory of human genetics in Mexico. The Infinium HTS protocol has also been used and described in previous works [16]. All the participants signed an informed consent for the present study and their personal data was anonymized.

### Study population, variant selection, clinical variant mining and calculation of allele frequencies

Genotype data from 1754 Mexican participants without a CF or a CFTR-RD diagnosis was initially integrated into the cohort. The data was filtered using PLINK [17], along with R and bash computational processes following internationally established quality control (QC) methods [18, 19]. Filters at the sample and variant levels were established using recommended parameters in the literature [18]. According to previous studies, only individuals with low heterozygosity (F) and low coefficients of relationship (π) were included using appropriate thresholds (|F| < 2σ, π < 0.125) as inclusion parameters [18, 19]. After applying the QC filters, data from only 1455 individuals met the inclusion criteria. From the complete microarray data (669,673 variants), only the genotypes of 361 variants within the regions comprising the *CFTR* gene, including its exons, introns and the 5′ and 3′ regions, were included for further analysis. These include pathogenic and non-pathogenic variants in the *CFTR* gene. Clinical classifications of the studied *CFTR* variants were exclusively extracted from the ClinVar database [20, 21]. All of them are related to CF and CFTR-RD for the 361 variants. Minor allele frequencies (MAF) were calculated for these variants in this population.

### Comparative analysis of allele frequencies with populations sampled by gnomAD

Minor allele frequencies (MAFs) of human variants from eight world populations in the *CFTR* gene were calculated from the public VCF data of gnomAD [22, 23]. The MAFs of the *CFTR* gene variants from gnomAD populations were compared with the MAFs of the same variants from the Mexican population. Only 110 out of 361 variants of this study were found in the gnomAD data. Pearson chi-square tests were calculated on these variants in Rstudio version 1.2.1 and R version 4.0.2. to obtain those with significant differences between the Mexican and eight populations from gnomAD.

## Results

**Fig. 1:**
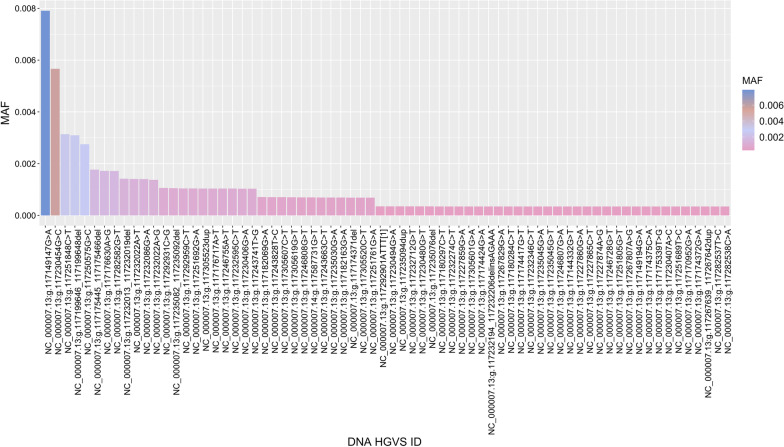
Frequency of pathogenic and likely pathogenic *CFTR* variants in a Mexican population. The horizontal axis shows the DNA HGVS ID of the variants, the vertical axis shows the minor allele frequency (MAF) for the pathogenic and likely pathogenic variants. Only variants with MAFs greater than zero are shown. All the pathogenic and likely-pathogenic variants have low frequencies (MAF < 0.01) compared to benign variants or variants with uncertain significance (MAF > 0.01)

**Fig. 2:**
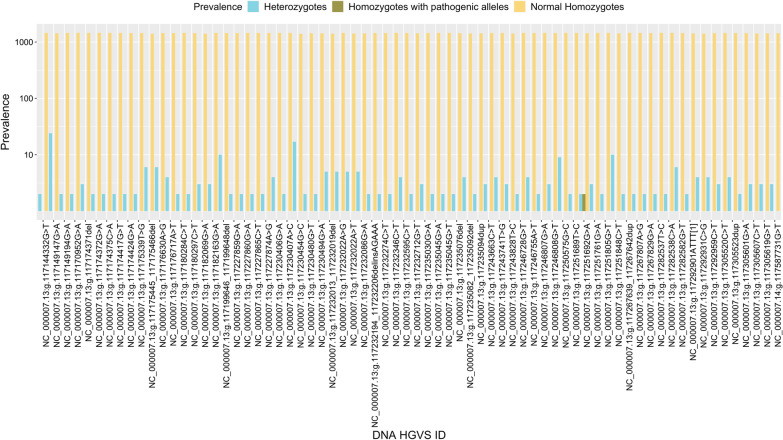
Prevalence of homozygotes and heterozygotes of the studied *CFTR* pathogenic variants. The vertical axis in log scale shows the prevalence of homozygotes/heterozygotes in the studied Mexican population, the horizontal axis shows the variant DNA HGVS ID. As expected, most of the pathogenic alleles are distributed in heterozygotes except for a single variant, from which an undiagnosed homozygote was detected

The minor allele frequencies (MAFs) calculated for the 361 studied variants are presented in Additional file 1: Table S1. Only the pathogenic and likely pathogenic MAFs are shown in Fig. 1, these variants have a low frequency in the Mexican population (MAF < 0.01); while the benign, likely benign and variants of uncertain significance have higher allele frequencies (MAF > 0.01). The most common pathogenic *CFTR* variants in the Mexican cohort correspond to the NC_000007.13:g.117149147G>A, NC_000007.13:g.117230454G>C, NC_000007.13:g.117251848C>T and NC_000007.13:g.117199646_117199648del with frequencies of 0.00790, 0.00566, 0.00314 and 0.00309, respectively. Figure 2 shows the prevalence of heterozygotes and homozygotes observed in the analyzed Mexican population for the pathogenic and likely pathogenic variants. It is not surprising that most of the pathogenic variants are distributed in heterozygotes in the open Mexican population; except for NC_000007.13:g.117251692G>A, from which a single undiagnosed homozygote was detected with no clinical reports of CF or CFTR-RD. We did not observe compound heterozygotes in the studied population. Additional file 1: Table S1 contains the DBSNP ID, the HGVS ID on the DNA level, the HGVS ID on the protein level, Variation type, clinical classifications, associated disorders (CF, CFTR-RD, etc.), and prevalence of heterozygotes and homozygotes of the 361 studied *CFTR* variants in the Mexican population.

The comparative analysis of the MAFs between populations was performed among 110 variants of the 361 studied in accordance with the gnomAD data. The gnomAD populations used for this comparative analysis are shown in Table 1, as well as the number of *CFTR* variants with significant differences with respect to the Mexican population. As expected, the Latin American population was the most similar to this cohort, while the Northwest European population and the Ashkenazi Jews displayed several variants with significant differences. In total, 48 out of the 110 compared variants showed significant differences between the Mexican and other populations. Some of these 48 variants exhibit differences multiple times on different populations during the whole comparative analysis, indicating possible specific population and genomic dynamics involving the *CFTR* gene. Additional file 2: Table S2 contains the results of the comparative analysis for the significantly different variants. There are some variants that are repeated several times during the comparison between the Mexican and other populations. For example, the NC_000007.13:g.117232022A>G variant displayed significantly different allele frequencies during the comparison between Mexican-Northwestern European populations, Mexican-African populations and Mexican-Estonian populations. This particular variant has a likely pathogenic classification on ClinVar.

**Table 1:** Statistical comparative analysis of *CFTR* variant frequencies between populations

Database	Population	Number of different variants
gnomAD	Latin Americans	12/110
gnomAD	East Asians	13/110
gnomAD	Finnish	17/110
gnomAD	Southern Europeans	19/110
gnomAD	Africans	19/110
gnomAD	Estonians	21/110
gnomAD	Ashkenazi Jews	22/110
gnomAD	Northwestern Europeans	25/110
This study	Mexicans	0/110

The first column describes the database of origin, the second one the target population of the comparison, and the third one shows the number of variants with significant differences against the Mexican population. The last row is a control of the analysis

GnomAD: https://gnomad.broadinstitute.org/. Last access date: August 15th, 2021

## Discussion

Since we selected all *CFTR* variants contained in the microarray data, the variants of this study include different clinical classifications ranging from benign to pathogenic, others with uncertain clinical significance or no available clinical information. From this data, an important subset are the pathogenic and likely pathogenic variants that are of interest for the clinical and epidemiological study of CF [5, 6, 10]. As it has been previously reported, we detected that one the most common CF pathogenic variant in the Mexican population corresponds to NC_000007.13:g.117199646_117199648del. However, we also found that the NC_000007.13:g.117149147G>A, NC_000007.13:g.117230454G>C and NC_000007.13:g.117251848C>T variants have higher frequencies in this cohort. The first and second variants have similar frequencies in other populations, while the third one has a lower frequency in other populations. It is reported that NC_000007.13:g.117149147G>A induces pancreatic disease and CFTR-RD, and NC_000007.13:g.117251848C>T reduces the activity of the encoded CFTR protein by 10–15%. Causing CF cases when combined with other pathogenic variants [24]. On the other hand, the NC_000007.13:g.117230454G>C variant has been associated with congenital bilateral absence of vas deferens, hereditary pancreatitis, classical and non-classical CF forms [25, 26]. In fact, the NC_000007.13:g.117230454G>C missense substitution was originally listed as a neutral polymorphism however, later studies detected this variant in patients with classical CF [26] and in patients with a clinical disease in a subgroup of the organ systems [27]. These non-classical CF forms, including late-onset pulmonary disease, congenital bilateral absence of vas deferens, and idiopathic pancreatitis frequently show a genetic diagnostic challenge for the unclear genotype–phenotype correlation [27].

In the comparative analysis between populations, we found that the majority of the *CFTR* allele frequencies followed similar tendencies to those reported by gnomAD. The gnomAD populations with the least significant differences to the Mexicans were the Latin Americans (from the US) and East Asians. On the other hand, the greatest significant differences were found when comparing this cohort with the European and African populations. We also observed that *CFTR* variants with higher frequencies (MAF > 0.01), namely those with a benign, unknown or with uncertain significance, display statistical differences among several world populations; while low frequency variants (MAF < 0.01) with pathogenic or likely pathogenic classification, displayed statistical differences between fewer populations. This analysis demonstrates the existence of significant epidemiological differences in the prevalence of *CFTR* variants between populations of different ethnicities and highlights the importance of this knowledge to improve the diagnosis and treatments of CF patients.

Finally, the growing knowledge of etiology and pathogenesis of CF has motivated the development of new pharmacotherapeutic strategies aimed at correcting gene dysfunction, which is why genotyping of CF patients also acquires importance for the future application of allele-specific therapy. An example of this is the FDA approval of Ivacaftor, a CFTR protein potentiator which increases channel opening time and chloride flux, mainly effective in patients with the NC_000007.13:g.117227860G>A variant [28]. Additionally, there are other available drugs like correctors (i.e. elexacaftor and tezacaftor) for other kinds of *CFTR* alleles (i.e. NC_000007.13:g.117199646_117199648del) [29, 30]. In practice, medical treatments of CF patients use combinations of different families of drugs including *CFTR* potentiators/correctors, but also antibiotics, anti-inflammatory medications, bronchodilators, etc. It is not surprising that as medical research advances, new pharmacogenomic alleles are discovered to improve drug treatments for CF patients.

## Conclusions

Studies of *CFTR* variants involved in CF play an important role in the research, diagnosis, surveillance and therapeutic development for this genetic disorder. The results of this study indicated that the Mexican population has similarities with the Latin American population from the US in the prevalence of *CFTR* variants. It also revealed significant differences with European and African populations. Epidemiologically, our data revealed the existence of other frequent as well as rare CF variants, and it will help to determine the prevalence of the CF etiology in Mexico. This project provides regional data on genetic variants in the *CFTR* gene for the national and international community that could help develop molecular diagnostic screening tests to optimize medical care of CF patients in Mexico.

## Supplementary Information

**Additional file 1: Table S1.** Minor allele frequencies (MAF) of the 361 genotyped CFTR variants in a Mexican population. The table shows dbSNP ID, DNA HGVS ID, PROTEIN HGVS ID, Variant type, Clinical significance according to ClinVar, Minor Allele Frequency (MAF), Number of homozygotes and heterozygotes for the variant, and Disorders associated with the variant. The data is in descending order with respect to the allele frequency calculated for the Mexican population.

**Additional file 2: Table S2.** Significant results of the comparative analysis for the CFTR allele frequencies between a Mexican population and gnomAD populations. Description of data: Only variants with significant differences to the Mexican frequencies are shown (*p* values < 0.05). The table contains dbSNP ID, DNA HGVS ID, PROTEIN HGVS ID, MAF for each population, chi-square value, p-value and the clinical significance of each variant.

## Data Availability

The data related to the allele frequencies of the *CFTR* variants in the Mexican population, including the prevalence of homozygotes and heterozygotes and the comparative analysis is available in the additional information files. The raw data and datasets analyzed during the current study are available from the corresponding author on reasonable request.

## Abbreviations

CF: Cystic fibrosis
CFTR: Cystic fibrosis transmembrane conductance regulator
CFTR-RD: CFTR-related disorders
QC: Quality control
MAF: Minor allele frequencies
